# Parapapillary choroidal microvascular density in acute primary angle-closure and primary open-angle glaucoma: an optical coherence tomography angiography study

**DOI:** 10.1136/bjo-2021-321022

**Published:** 2022-07-13

**Authors:** Yanin Suwan, Masoud Aghsaei Fard, Nantinee Vilainerun, Purit Petpiroon, Apichat Tantraworasin, Chaiwat Teekhasaenee, Robert Ritch, Rahele Kafieh, Sahar Hojati, Wasu Supakontanasan

**Affiliations:** 1 Ophthalmology, Ramathibodi Hospital, Bangkok, Thailand; 2 Ophthalmology, Farabi Eye Hospital, Tehran, Iran (the Islamic Republic of); 3 Ophthalmology, Chulabhorn hospital, Chulabhorn Royal Academy, Bangkok, Thailand; 4 Surgery and Clinical Epidemiology and Clinical Statistic Center, Faculty of Medicine, Chiang Mai University, Chiang Mai, Thailand; 5 Clinical Surgical Research Center, Chiang Mai University, Chiang Mai, Thailand; 6 Einhorn Clinical Research Center, New York Eye and Ear Infirmary of Mount Sinai, New York City, New York, USA; 7 Medical Image and Signal Processing Research Center, School of Advanced Technologies in Medicine, University of Medical Sciences, Isfahan, Iran

**Keywords:** Glaucoma, Imaging, Optic Nerve, Choroid, Angle

## Abstract

**Back ground/aims:**

To determine whether parapapillary choroidal microvasculature (PPCMv) density, measured by optical coherence tomography angiography, differed between acute primary angle-closure (APAC), primary open-angle glaucoma (POAG) and controls.

**Methods:**

This is a prospective, cross-sectional, observational study. Data from 149 eyes from two academic referral centres were analysed. Automated PPCMv density was calculated in inner and outer annuli around the optic nerve region in addition to the peripapillary superficial vasculature, using customised software. A generalised estimating equation was used to compare vessel densities among groups, adjusted for confounders.

**Results:**

Data from 40 eyes with APAC, 65 eyes with POAG and 44 eyes in healthy controls were gathered and analysed. Global radial peripapillary capillary densities were reduced in eyes with APAC and POAG compared with controls (p=0.027 and 0.136, respectively). Mean outer annular PPCMv density in the POAG group was lower vs the APAC group by 3.6% (95% CI 0.6% to 6.5%) (p=0.018) in the multivariable model adjusted for confounders. The mean difference in inner and outer superior PPCMv between the POAG and APAC groups was 5.9% and 4.4% (95% CI 1.9% to 9.9% and 1.0% to 7.7%, respectively; both p<0.010). Furthermore, POAG and APAC groups both had significantly lower PPCMv compared with controls (both, p<0.001).

**Conclusions:**

While superficial peripapillary vessels were affected to similar degrees in POAG and APAC, PPCMv drop-out was greater with POAG versus APAC, suggesting that choroidal vessel density may be affected to a lesser extent following an acute increase in intraocular pressure before glaucoma develops.

**What is already known on this topic?**

- Microvascular alteration was detected at superficial peripapillary area after APAC episode.

**What this study adds?**

- Parapapillary choroidal microvascular density decreased in both APAC and POAG but to a different extent.

**How this study might affect research, practice or policy?**

- This study provides quantitative analysis of choroidal microvascular disturbance.

Primary angle-closure glaucoma (PACG) is more prevalent in Asian eyes compared with white populations.^1^ Although PACG is only approximately one-third as prevalent as open-angle glaucoma, PACG causes three times more blindness than primary open-angle glaucoma (POAG).^2^ Acute primary angle closure (APAC) is a crucial presentation of PACG characterised by severe and abrupt intraocular pressure (IOP) elevation following sudden blockage of the aqueous outflow. This brisk and significant increase in IOP damages the optic nerve both structurally and functionally. The parapapillary choroid, which supplies the prelaminar tissue and the lamina cribrosa of the optic nerve head, is the most susceptible to obliteration by elevated IOP.^3 4^ In addition to increased IOP, ‘primary’ reduced ocular blood flow also plays a significant role in the pathogenesis of glaucoma.^5 6^ Primary vascular insufficiency as a cause of glaucomatous optic neuropathy, other than mechanical damage, has been studied in POAG,^7–9^ normal tension glaucoma^10^ and PACG.^11–13^

Optical coherence tomography angiography (OCTA) enables visualisation of the retinal and choroidal microvasculature and has shown a reduced superficial radial peripapillary capillary (RPC) density and deep choroidal microvasculature drop-out in POAG and PACG eyes.^13–19^ Choroidal microvasculature drop-out is significantly lower in PACG than in POAG, suggesting a role for primary choroidal ischaemic injury in the pathogenesis of POAG.^20^ However, to our knowledge, studies evaluating deep choroidal microvasculature damage following acute IOP elevations in APAC are lacking. Furthermore, while both primary and/or secondary parapapillary vascular rarefaction might be present in POAG and APAC, the contribution of primary vs secondary vascular drop-out in each disease has not been fully determined. Because both elevated IOP and primary vascular damage could lead to vessel drop-out, APAC evaluation provides the opportunity to evaluate the short-term effect of high IOP on deep parapapillary vessel density without considering the effect of chronic primary vascular damage, as seen in POAG.

Therefore, this study aimed to compare choroidal vessel density among patients with APAC and POAG, and controls using automated image processing for choroidal microvessel quantification to investigate the pathogenesis of vascular damage in different types of glaucoma.

## Materials and methods

This prospective, cross-sectional, observational study was conducted from February 2021 to December 2021 in Ramathibodi Hospital and Farabi Eye Hospital. This study was Health Insurance Portability and Accountability Act compliant and adhered to the tenets of the Declaration of Helsinki.

### Participants

This study included participants with treated APAC or POAG of varying severities, and healthy controls. Inclusion criteria for all participants were age ≥20 years and best-corrected visual acuity (BCVA) of 20/40 or better. APAC was defined according to the following criteria^12^: (1) presence of at least two of the following symptoms: periocular pain or headache, nausea and/or vomiting, decreased vision and history of rainbow-coloured halos around light; (2) documentation of presenting IOP ≥30 mm Hg using Goldmann applanation tonometry during the acute episode; (3) presence of at least four of the following slit-lamp biomicroscopic findings: ciliary injection, corneal epithelial oedema, fixed mid-dilated pupil, glaukomflecken, and shallow peripheral anterior chamber; (4) presence of invisible posterior trabecular meshwork of >270° on gonioscopy and (5) IOP <21 mm Hg after medication, laser, anterior chamber paracentesis or cataract extraction.

POAG was defined according to the following criteria: presence of (1) a glaucomatous-appearing optic nerve (neuroretinal rim thinning or notching, and retinal nerve fibre layer (RNFL) defects) as documented by glaucoma experts; (2) an open angle on gonioscopy; (3) presence of glaucomatous visual field (VF) defect and (4) circumpapillary RNFL (cpRNFL) thinning on OCT outside the 95% CI of normal distribution corresponding to optic disc appearance and VF defect. A glaucomatous VF defect was defined as the presence of a cluster depressed >3 points at a 5% level of significance on the pattern deviation plot, with one or more of these points depressed at a 1% level while excluding points on the edge of the field; a glaucoma hemifield test result outside the normal limits; and abnormal pattern SD with a probability value of <5%. All VF defects were detected in at least two consecutive baseline VF tests and were consistent with a glaucomatous pattern.

The control group was defined as follows: subjects with IOP <22 mm Hg, no history of increased IOP, no history of diabetes mellitus, absence of glaucomatous disc neuropathy, open angle, no VF defects and no evidence of pseudoexfoliation material on the anterior lens capsule or pupillary margin after mydriasis on slit-lamp biomicroscopy in either eye.

The exclusion criteria for all groups were the presence of eyes with recurrent/subacute angle closure, secondary angle closure, history of ocular surgery other than uncomplicated cataract surgery, a spherical refraction greater than ±6 diopters (D) and cylinder correction greater than ±3 D, ocular media opacities that prevented good quality scans, vitreoretinal diseases or nonglaucomatous optic neuropathy, cardiovascular disease apart from systemic hypertension, diabetes mellitus, and ocular or systemic steroid use.

### Clinical examinations

All participants underwent complete ophthalmic examinations within 6 months of OCTA imaging, including BCVA (Snellen), slit-lamp biomicroscopy, IOP measurement using Goldmann applanation tonometry, gonioscopy, dilated fundus examination using a 78-D noncontact slit-lamp lens (Volk Optical, Mentor, Ohio, USA), and axial length measurement using A-scan biometry (IOL Master; Carl Zeiss Meditec, Dublin, California, USA) were performed. VF examinations were performed using the Humphrey Visual Field Analyzer II, model 740 (Zeiss Humphrey Systems, Dublin, California, USA) using a 24-2 Swedish interactive thresholding algorithm standard protocol. The participants’ medical histories, including systemic blood pressure and systemic and ophthalmic medications, were reviewed and recorded. Both eyes of each participant were imaged and analysed.

### Spectral-domain optical coherence tomography and OCTA

All subjects underwent OCT and OCT-A imaging after the acute attack had fully resolved, using the AngioVue imaging system (AngioVue Software V.2011.1.1.151; OptoVue, Fremont, California, USA). A standard circumpapillary scan was used to measure cpRNFL thickness, and the average and each quadratic cpRNFL value were recorded.

For OCTA, superficial and choroidal blood flow information was obtained at the level of the RPC and choroid in a 4.5×4.5 mm scan centred on the optic disc. We used en face imaging and employed customised MATLAB software (MathWorks, Natick, Massachusetts, USA). We calculated peripapillary perfused capillary density (PCD) after removing the densities of large retinal vessels and parapapillary choroidal microvasculature (PPCMv) density after removing the shadows of the large retinal vessels and ignoring the information inside the disc, as described previously.^17 21 22^ Briefly, for PCD, a 3.45 mm diameter outer circle was placed concentric to the inner 1.95 mm circle, producing an annular region of interest (ROI) with a width of 0.75 mm. Whole-image vessel density (global) and annular peripapillary density and its four quadrant vessel density values were reported. For PPCMv, an inner circle was automatically placed around the disc location, and a second middle circle and final third circles were placed homocentrically with a diameter of 1 mm and 2 mm greater than the inner circle, respectively. Annular PPCMv density values with widths of 0.5 mm and their superior and inferior halves were calculated.^17^

After reviewing the raw OCT images, images with significant artefacts, such as background noise, signal strength index <40, residual motion artefacts visible as irregular vessel pattern or disc boundary, and segmentation errors, were excluded.

### Statistical analysis

The Shapiro-Wilk test was used to assess the distribution of numerical data. Gaussian-distributed variables were described as the mean and SD, and non-Gaussian-distributed variables were described as median and IQR.

Categorical variables were compared using the χ^2^ test. We used a marginal model of a generalised estimating equation adjusted for age, sex, axial length, VF mean deviation (VF MD) and average cpRNFL thickness to evaluate the differences in the variables between groups. Mixed-effect multilevel regression stratified by laterality was performed to assess the association between PCD and PPCM among the three groups in all study eyes. Statistical analysis was performed with the SPSS software (V.22.0; IBM). P values<0.05 were considered significant.

## Results

### Study population

Initially, 158 eyes of 112 patients met the inclusion criteria for this study. Among the 158 eyes, 1 AAC eye and 1 POAG eye were excluded because of unacceptable OCTA image quality. Seven AAC eyes with glaucomatous VF defect were also excluded. Therefore, 40 eyes with APAC (mean (SD) age, 62 (7) years; 30 (75%) women); 65 eyes with POAG (59 (12) years; 34 (52%) women); and 44 eyes in the healthy controls (65 (6) years;17 (39%) women) were included. Four patients had bilateral APAC.

There were significant differences between the groups for age, sex, axial length, VF MD, VF pattern SD and average and quadratic cpRNFL (p for all=0.003) (table 1). The period from acute attack onset to OCTA imaging was (median (IQR)) 265 (138–1562) days.

**Table 1:** Demographic, visual field and circumpapillary retinal nerve fibre layer (cpRNFL) thickness characteristics of the participants

Variables	APAC N=40	POAG N=65	Controls N=44	P value			
				Among* 3 groups	APAC† versus controls	POAG† versus controls	APAC† versus POAG
Age, year, (mean±SD)	62±7	59±12	65±6	0.001	0.396	0.001	0.161
Female, n (%)	30 (75.00)	34 (52.31)	17 (38.64)	0.003			
Axial length, mm (mean±SD)	22.71±1.16	23.92±1.04	22.98±1.18	<0.001	0.815	0.001	<0.001
VF MD, dB (median (IQR))	−4.09 (−4.52 to 2.78)	−5.02 (−8.62 to 3.02)	−1.80 (−3.67 to 0.11)	<0.001	0.008	<0.001	0.019
VF PSD, dB (median (IQR))	2.63 (1.90–5.00)	6.66 (3.36–11.63)	2.14 (1.98–3.43)	<0.001	0.147	<0.001	<0.001
Visual Field Index, % (median (IQR))	98 (96–99)	89.5 (79–96)	–	<0.001	–	–	<0.001
Average cpRNFL, μm (median (IQR))	98 (87–103)	83.5 (71.5–95.0)	102.5 (95–108)	<0.001	0.013	<0.001	<0.001
Superior cpRNFL, μm (median (IQR))	116 (93–127)	108 (92.5–119.25)	127.5 (117–141)	<0.001	<0.01	<0.001	0.051
Inferior cpRNFL, μm (median (IQR))	112.5 (92–126)	88.5 (70.75–107.05)	132.5 (125–145)	<0.001	0.003	<0.001	0.008
Nasal cpRNFL, μm (median (IQR))	75. (66–88.5)	70.25 (58.25–78.75)	86 (79.5–96)	<0.001	<0.001	<0.001	0.003
Temporal cpRNFL, μm (median (IQR))	76 (66.5–84)	69.25 (59.75–75)	79.5 (71.5–84)	<0.001	0.191	<0.001	0.001

The participants’ eye baseline clinical data per group are reported as mean±SD and median with IQR. Categorical variables: χ^2^ test.

*P values represent comparisons between APAC, POAG and control groups using ANOVA.

†P value represent pairwise comparison between each groups using Bonferroni correction or Dunn’s test.

ANOVA, analysis of variance; APAC, acute primary angle closure; MD, mean deviation; POAG, primary open-angle glaucoma; PSD, pattern SD; VF, visual field.

### Peripapillary perfused capillary densities

Global, annular and quadratic (except for temporal) PCDs were significantly different between the three groups (p=0.007, except for the nasal and temporal quadrants (p=0.061 and 0.073, respectively), in the univariable analysis (table 2). Multivariable linear regression using a generalised estimating equation showed no significant difference in global, annular and quadratic PCD values between the experimental groups and the controls (p>0.081), except for global, superior and inferior quadrants (p<0.027), when adjusted for age, sex, axial length, cpRNFL and VF MD (table 3). There was no significant difference in global, annular and all quadratic PCD values between POAG and APAC (p>0.201).

**Table 2:** Comparisons of global, annular and quadratic PCDs among the APAC, POAG and control groups

PCD, % mean (SD)	APAC N=40	POAG N=65	Controls N=44	P value			
				Among* 3 groups	APAC† versus controls	POAG† versus controls	APAC† versus POAG
Global (mean±SD)	21.97±7.52	20.41±8.70	27.25±5.33	<0.001	0.009	<0.001	0.975
Annular (mean±SD)	24.26±8.26	24.79±10.35	29.93±5.60	0.007	0.016	0.015	1.000
Superior (median (IQR))	20.32 (14.09–24.74)	20.45 (12.85–27.41)	29.67 (25.45–31.65)	<0.001	<0.001	<0.001	0.308
Inferior (median (IQR))	19.73 (15.95–25.88)	20.18 (12.26–28.65)	29.18 (25.02–34.09)	<0.001	<0.001	<0.001	0.327
Nasal (median (IQR))	20.90 (16.11–27.11)	24.08 (17.23–36.61)	26.51 (22.71–29.83)	0.061	0.010	0.173	0.049
Temporal mean±SD)	30.16±9.82	28.55±12.41	33.56±7.40	0.073	0.493	0.068	1.000

*P values represent comparisons between APAC, POAG, and control groups using analysis of variance analysis.

†P value represent pairwise comparison between each groups using Bonferroni correction or Dunn’s test.

APAC, acute primary-angle closure; PCD, peripapillary perfused capillary density; POAG, primary open-angle glaucoma.;

**Table 3:** Mean difference in global, annular and quadratic PCDs between the three groups

PCD, %, mean (SD)	Mean difference	95% CI	P value
Global			
Control	Reference		
POAG	−3.46	−7.98 to 1.09	0.136
APAC	−4.66	−8.40 to −0.52	0.027
APAC versus POAG	−1.02	−5.70 to 3.67	0.671
Annular			
Control	Reference		
POAG	−0.77	−6.03 to 4.49	0.774
APAC	−4.06	−8.62 to 0.50	0.081
APAC versus POAG	−3.29	−8.68 to 2.10	0.232
Superior			
Control	Reference		
POAG	−4.31	−9.17 to 0.55	0.082
APAC	−6.89	−11.17 to −2.61	0.002
APAC versus POAG	−2.58	−7.69 to 2.52	0.322
Inferior			
Control	Reference		
POAG	−4.06	−10.00 to 1.88	0.181
APAC	−5.84	−10.97 to −0.70	0.026
APAC versus POAG	−1.78	−7.81 to 4.26	0.564
Nasal			
Control	Reference		
POAG	2.61	−4.00 to 9.22	0.438
APAC	−1.86	−7.62 to 3.91	0.528
APAC versus POAG	−4.47	−11.32 to 2.38	0.201
Temporal			
Control	Reference		
POAG	−1.18	−7.08 to 4.72	0.696
APAC	−3.60	−8.94 to 1.74	0.186
APAC versus POAG	−2.43	−8.81 to 3.96	0.456

Analysed by the marginal model of a generalised estimating equation adjusted for age, sex, axial length, VF MD and circumpapillary retinal nerve fibre layer thickness.

APAC, acute primary angle closure; MD, mean deviation; PCD, peripapillary perfused capillary density; POAG, primary open-angle glaucoma; VF, visual field.

### Parapapillary choroidal microvasculature

**Figure 1:**
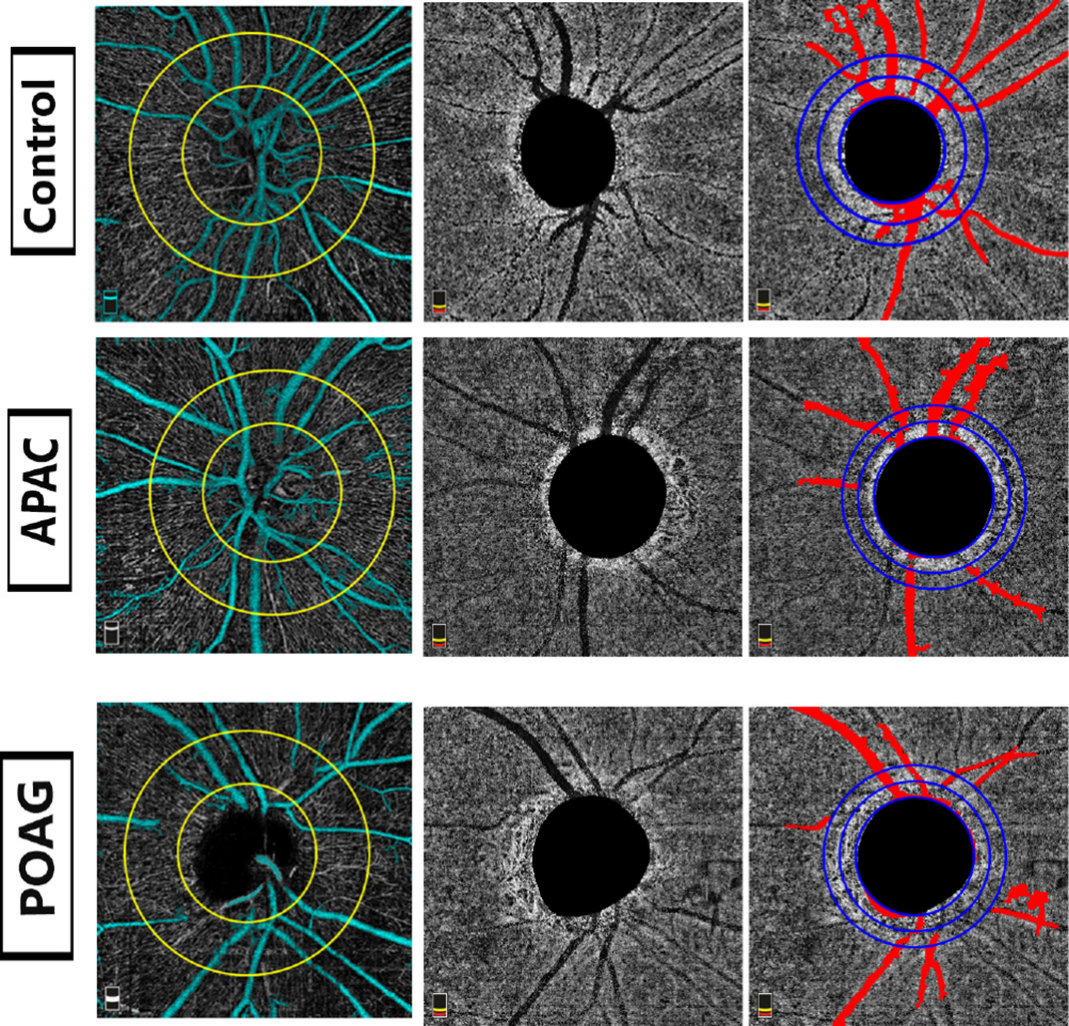
Parapapillary choroidal microvasculature in acute primary-angle closure (APAC) glaucoma, primary open-angle closure (POAG) glaucoma and controls. Left: OCTA images of peripapillary superficial vessel density after removing large vessels (cyan); middle, OCTA of deep parapapillary vessel images; (right) OCTA of deep parapapillary microvasculature after removing retinal vessels shadow (red) in three study eyes. OCTA, optical coherence tomography angiography.

There were significant differences in inner and outer annuli and inferior and superior hemifield PPCMv densities between the three groups in the univariable analysis (all p<0.001). There were significant differences in pairwise comparison between each group (all p=0.001), except for inner inferior hemifield (p=0.055) (table 4). OCTA images in grey scale from each group are shown in figure 1. Multivariable linear regression using a generalised estimating equation showed significant differences in inner and outer annuli and inferior and superior hemifield PPCMv densities between the experimental and control groups (p<0.001) when adjusted for age, sex, axial length, cpRNFL and VF MD (table 5). There were significant differences in PPCMv densities between POAG and APAC in all ROIs except for the inner annulus, and inner and outer inferior hemifields (p=0.052, 0.445 and 0.078, respectively). In addition, there was a trend towards decreasing PPCMv density from control to APAC to POAG groups.

**Table 4:** Comparisons of global, annular and all region of interest PPCMv values among the APAC, POAG and control groups

PPCMv	APAC N=51	POAG N=66	Controls N=46	P value			
				Among* 3 groups	APAC† versus Controls	POAG† versus Controls	APAC† versus POAG
Inner annulus (median (IQR))	12.1 (8.9–15.6)	7.7 (4.7–11.4)	24.5 (19.8–29.3)	<0.001	<0.001	<0.001	0.001
Inner hemisuperior (median (IQR))	12.7 (9.4–15.9)	6.6 (3.9–10.4)	25.5 (21.6–30.1)	<0.001	<0.001	<0.001	<0.001
Inner hemi-inferior (median (IQR))	11.0 (8.8–16.0)	9.8 (5.3–12.7)	23.8 (19.1–27.9)	<0.001	<0.001	<0.001	0.055
Outer annulus (median (IQR))	9.7 (6.4–13.0)	5.6 (4.0–8.0)	18.3 (15.6–21.9)	<0.001	<0.001	<0.001	<0.001
Outer hemi-superior (median (IQR))	10.1 (6.5–13.1)	4.8 (2.6–8.3)	19.4 (16.2–23.1)	<0.001	<0.001	<0.001	<0.001
Outer hemiinferior (median (IQR))	9.4 (7.2–12.9)	6.1 (4.5–8.7)	16.9 (13.9–21.2)	<0.001	<0.001	<0.001	<0.001

*P values represent comparisons between APAC, POAG and control groups using analysis of variance analysis.

†P value represent pairwise comparison between each groups using Dunn’s test.

APAC, acute primary-angle closure; POAG, primary open-angle glaucoma; PPCMv, parapapillary choroidal microvasculature.

**Table 5:** Mean difference in global, annular and all region of interest PPCMv values between the three groups

PPCMv	Mean difference	95% CI	P value
Inner annulus			
Control	Reference		
POAG	−16.5	−20.1 to −13.0	<0.001
APAC	−12.9	−15.9 to −9.9	<0.001
APAC vs POAG	3.6	−0.0 to 7.3	0.052
Inner hemisuperior			
Control	Reference		
POAG	−19.4	−23.4 to −15.4	<0.001
APAC	−13.5	−16.8 to −10.2	<0.001
APAC versus POAG	5.9	1.9 to 9.9	0.004
Inner hemi-inferior			
Control	Reference		
POAG	−14.1	−18.4 to −9.8	<0.001
APAC	−12.4	−16.0 to −8.7	<0.001
APAC versus POAG	1.7	−2.7 to 6.2	0.445
Outer annulus			
Control	Reference		
POAG	−12.5	−15.4 to −9.5	<0.001
APAC	−8.9	−11.4 to −6.4	<0.001
APAC versus POAG	3.6	0.6 to 6.5	0.018
Outer hemi-superior			
Control	Reference		
POAG	−14.4	−17.6 to −11.1	<0.001
APAC	−10.0	−12.8 to −7.3	<0.001
APAC versus POAG	4.4	1.0 to 7.7	0.010
Outer hemi-inferior			
Control	Reference		
POAG	−10.9	−14.2 to −7.7	<0.001
APAC	−8.0	−10.7 to −5.2	<0.001
APAC versus POAG	3.0	−0.3 to 6.3	0.078

Analysed by the marginal model of a generalised estimating equation adjusted for age, sex, axial length, VF MD, and circumpapillary retinal nerve fibre layer thickness.

APAC, acute primary angle closure; MD, mean deviation; POAG, primary open-angle glaucoma; PPCMv, parapapillary choroidal microvasculature; VF, visual field.

There was no significant factor associated with inner and outer annuli PPCMv densities, when analysed using mixed-effect multilevel regression stratified by laterality (p>0.071) (table 6).

**Table 6:** Determinants of parapapillary choroidal microvasculature

PPCMv	Mean difference	95% CI	P value
Inner annulus			
Age	0.002	−0.001 to 0.004	0.079
Axial length	−0.010	−0.022 to 0.001	0.081
Female	−0.014	−0.052 to 0.024	0.467
VFMD	0.002	−0.001 to 0.006	0.240
Outer annulus			
Age	0.001	−0.001 to 0.003	0.101
Axial length	−0.005	−0.013 to 0.003	0.261
Female	−0.011	−0.040 to 0.018	0.449
VFMD	0.002	−0.001 to 0.005	0.071

Analysed by mixed-effect multi-level regression stratified by laterality.

MD, mean deviation; PPCMv, parapapillary choroidal microvasculature; VF, visual field.

## Discussion

In this study, the inner, outer and both superior and inferior hemispheric PPCMv values, using automated customised software, represented PPCMv drop-out in both POAG and APAC eyes. We also demonstrated a trend towards decreasing PPCMv density from control to APAC to POAG groups. However, superficial peripapillary vessel density values did not differ between APAC and POAG.

For APAC eyes, the pathogenesis of glaucoma differs from other types of glaucoma; therefore, microvascular changes may also differ. Both mechanical compression of the optic disc from severe and abrupt IOP elevation in APAC as well as vascular dysregulation are involved in glaucomatous damage, which differs from POAG. Retinal blood flow remains normal over a wide range of elevated IOP because of autoregulation. The critical IOP, which is the point at which blood flow begins to decline, varies significantly from 5 to 40 mm Hg depending on the measurement method, individual variation and the underlying reason inducing the IOP elevation.^23–27^ In eyes with APAC, IOP elevates dramatically higher than reported critical IOP and has markedly detrimental effects on ocular blood flow. Previous studies have reported reduced peripapillary superficial retinal vessel density in APAC eyes compared with that in primary angle-closure suspect eyes,^4^ the fellow eye,^11 28^ POAG^12^ and controls.^12^ An early progressive loss of RPC density after the onset of APAC has been reported, even when the RNFL was in the edematous phase.^29^ In this study, we found a comparable decrease in superficial vessel density values between POAG and APAC, similar to a previous study.^12^ While loss of superficial vessels in APAC might be due to a sudden elevation in IOP, POAG eyes show vessel loss due to chronic cpRNFL loss.^12^

Recently, the parapapillary choroidal circulation, supplied by the short posterior ciliary arteries, is of particular interest in glaucoma as a potential surrogate marker for the perfusion of the deep optic nerve head structures. Previous studies reported a significantly lower prevalence of choroidal microvascular drop-out in PACG compared with POAG, especially in early glaucoma.^20 30^ In this study, we found a significant difference in PPCMv density between APAC and POAG. Furthermore, there was a trend towards progressive PPCMv drop-out from control to APAC to POAG groups. Our study showed that in APAC eyes, choroidal microvasculopathy occurs even with a single episode of elevated IOP.

Therefore, the degree and pathogenesis of PPCMv drop-out differs in POAG and APAC. In APAC, decreased PPCMv density may be at least partly attributed to optic nerve damage by IOP elevation rather than a primary choroidal vasculopathy, as in POAG. In addition, it appears that the degree of IOP damage in the deep microvasculature in APAC is less than with a primary vasculopathy in POAG. In contrast to superficial vascular assessment, which showed a similar loss in both conditions, deep vessels are less affected owing to the high IOP in APAC.

Regarding the analysis of factors associated with changes in PPCMv, our study found no significant factor associated with inner and outer annuli PPCMv densities. Furthermore, our study contradicted the previous report in which axial length was a strong determinant and negatively correlated with peripapillary vessel density.^31–34^

This study has a number of limitations. First, the cross-sectional design precluded identification of a primary causative aetiology. Additionally, the metrics are only surrogate measures of blood flow, and their relationship with actual blood flow values remains to be elucidated. Furthermore, patients were not followed over time, and we could not determine disease progression. Second, the effect of antiglaucoma medications on vessel density in POAG and APAC groups was not evaluated. Third, visualisation of choroidal vasculature with current OCTA technology is limited by projection artefacts that may have caused under detection of PPCMv. Fourth, the small sample size limited the power of our findings.

In summary, this study provides a quantitative analysis of choroidal microvascular disturbance in APAC compared with POAG. Our findings suggest that PPCMv density decreases in both APAC and POAG but to a different extent.

The presence and location of focal laminar cribrosa (LC) defects and their temporal relationship with PPCMv density change in APAC is warranted in future studies because LC, deep retinal layers and the choroid share a common blood supply from the short posterior ciliary artery.^35–37^ Future studies evaluating the correlation of PPCMv with structures (cpRNFL and ganglion cell complex thickness) and function (VF MD) could provide a greater understanding of vascular structural change over time and might be useful to predict visual function prognosis.

## Data Availability

Data are available on reasonable request.
